# Supplementary material for: Modeling glioblastoma heterogeneity as a dynamic network of cell states
Source: Mol Syst Biol. 2021 Sep 16;17(9):e10105. doi: 10.15252/msb.202010105 (PMC8444284; doi:10.15252/msb.202010105)
Supplement: Supplementary file 5 — Source Data for Figure 3 [file MSB-17-e10105-s001.zip › Figure3A_sourcedata/GSEA_3065/nosigdb_state1.GseaPreranked.1622718409535/SUVA_OPC_PMID_31327527.html]

Details for gene set SUVA\_OPC\_PMID\_31327527[GSEA]

|  || Dataset | state1 |
| Phenotype | NoPhenotypeAvailable |
| Upregulated in class | na\_neg |
| GeneSet | SUVA\_OPC\_PMID\_31327527 |
| Enrichment Score (ES) | -0.80214703 |
| Normalized Enrichment Score (NES) | -2.3671741 |
| Nominal p-value | 0.0 |
| FDR q-value | 0.0 |
| FWER p-Value | 0.0 |
Table: GSEA Results Summary

  

Fig 1: Enrichment plot: SUVA\_OPC\_PMID\_31327527      
 Profile of the Running ES Score & Positions of GeneSet Members on the Rank Ordered List

  

| PROBE | GENE SYMBOL | GENE\_TITLE | RANK IN GENE LIST | RANK METRIC SCORE | RUNNING ES | CORE ENRICHMENT || 1 | DBI |  |  | 110 | 0.352 | 0.0155 | No |
| 2 | CNTN1 |  |  | 911 | 0.143 | -0.0549 | No |
| 3 | FABP5 |  |  | 1470 | 0.091 | -0.1047 | No |
| 4 | CA10 |  |  | 1674 | 0.077 | -0.1195 | No |
| 5 | LIMA1 |  |  | 1678 | 0.077 | -0.1140 | No |
| 6 | FGF12 |  |  | 1872 | 0.066 | -0.1286 | No |
| 7 | TNS3 |  |  | 1918 | 0.064 | -0.1283 | No |
| 8 | PSAT1 |  |  | 4318 | -0.001 | -0.3719 | No |
| 9 | RAB31 |  |  | 7256 | -0.057 | -0.6660 | No |
| 10 | CNP |  |  | 7303 | -0.058 | -0.6663 | No |
| 11 | RTKN |  |  | 7357 | -0.060 | -0.6672 | No |
| 12 | EPB41L2 |  |  | 8402 | -0.099 | -0.7657 | No |
| 13 | TMEM206 |  |  | 8669 | -0.115 | -0.7840 | No |
| 14 | LMF1 |  |  | 8699 | -0.117 | -0.7781 | No |
| 15 | PHYHIPL |  |  | 8937 | -0.137 | -0.7917 | Yes |
| 16 | FIBIN |  |  | 9036 | -0.145 | -0.7907 | Yes |
| 17 | THY1 |  |  | 9119 | -0.153 | -0.7874 | Yes |
| 18 | CADM2 |  |  | 9141 | -0.157 | -0.7777 | Yes |
| 19 | VCAN |  |  | 9151 | -0.158 | -0.7666 | Yes |
| 20 | SIRT2 |  |  | 9386 | -0.198 | -0.7753 | Yes |
| 21 | SERINC5 |  |  | 9510 | -0.228 | -0.7706 | Yes |
| 22 | NLGN3 |  |  | 9554 | -0.241 | -0.7567 | Yes |
| 23 | PCDHGC3 |  |  | 9588 | -0.255 | -0.7407 | Yes |
| 24 | FXYD6 |  |  | 9631 | -0.273 | -0.7244 | Yes |
| 25 | PLP1 |  |  | 9702 | -0.314 | -0.7077 | Yes |
| 26 | RNF13 |  |  | 9703 | -0.314 | -0.6839 | Yes |
| 27 | P2RX7 |  |  | 9755 | -0.360 | -0.6619 | Yes |
| 28 | ALCAM |  |  | 9818 | -0.493 | -0.6308 | Yes |
| 29 | PGRMC1 |  |  | 9826 | -0.516 | -0.5925 | Yes |
| 30 | SCD5 |  |  | 9838 | -0.576 | -0.5500 | Yes |
| 31 | OLIG1 |  |  | 9845 | -0.637 | -0.5024 | Yes |
| 32 | PTPRZ1 |  |  | 9869 | -0.918 | -0.4352 | Yes |
| 33 | PMP2 |  |  | 9871 | -0.982 | -0.3610 | Yes |
| 34 | TTYH1 |  |  | 9872 | -1.033 | -0.2827 | Yes |
| 35 | GPM6B |  |  | 9873 | -1.091 | -0.2001 | Yes |
| 36 | BCAN |  |  | 9878 | -1.175 | -0.1116 | Yes |
| 37 | SCRG1 |  |  | 9879 | -1.473 | 0.0000 | Yes |
Table: GSEA details [plain text format]

  

Fig 2: SUVA\_OPC\_PMID\_31327527: Random ES distribution      
 Gene set null distribution of ES for **SUVA\_OPC\_PMID\_31327527**
